# A novel phenolic derivative inhibits AHL-dependent quorum sensing signaling in *Pseudomonas aeruginosa*

**DOI:** 10.3389/fphar.2022.996871

**Published:** 2022-09-20

**Authors:** Giulia Bernabè, Giovanni Marzaro, Giuseppe Di Pietra, Ana Otero, Massimo Bellato, Anthony Pauletto, Melania Scarpa, Stefania Sut, Adriana Chilin, Stefano Dall’Acqua, Paola Brun, Ignazio Castagliuolo

**Affiliations:** ^1^ Department of Molecular Medicine, University of Padua, Padua, Italy; ^2^ Department of Pharmaceutical and Pharmacological Sciences, University of Padua, Padua, Italy; ^3^ Departamento de Microbioloxía e Parasitoloxía, Facultade de Bioloxía-CIBUS, Universidade de Santiago de Compostela, Santiago de Compostela, Spain; ^4^ Department of Information Engineering, University of Padua, Padua, Italy; ^5^ Laboratory of Advanced Translational Research, Veneto Institute of Oncology IOV—IRCCS, Padua, Italy

**Keywords:** biofilm, antibiotic resistance, virulence, quorum sensing inhibitors, molecular docking

## Abstract

Increasing antibiotic resistance and the decline in the pharmaceutical industry’s investments have amplified the need for novel treatments for multidrug-resistant bacteria. Quorum sensing (QS) inhibitors reduce pathogens’ virulence without selective pressure on bacteria and provide an alternative to conventional antibiotic-based therapies. *P. aeruginosa* uses complex QS signaling to control virulence and biofilm formation. We aimed to identify inhibitors of *P. aeruginosa* QS acting on acyl-homoserine lactones (AHL)-mediated circuits. Bioluminescence and qRT-PCR assays were employed to screen a library of 81 small phenolic derivatives to reduce AHL-dependent signaling. We identified GM-50 as the most active compound inhibiting the expression of AHL-regulated genes but devoid of cytotoxic activity in human epithelial cells and biocidal effects on bacteria. GM-50 reduces virulence factors such as rhamnolipids, pyocyanin, elastase secretion, and swarming motility in *P. aeruginosa* PAO1 laboratory strain. By molecular docking, we provide evidence that GM-50 highly interacts with RhlR. GM-50 significantly improved aztreonam-mediated biofilm disruption. Moreover, GM-50 prevents adhesion of PAO1 and inflammatory damage in the human A549 cell line and protects *Galleria mellonella* from PAO1-mediated killing. GM-50 significantly reduces virulence factors in 20 *P. aeruginosa* clinical isolates from patients with respiratory tract infections. In conclusion, GM-50 inhibits AHL-signaling, reduces virulence factors, enhances the anti-biofilm activity of aztreonam, and protects *G. mellonella* larvae from damage induced by *P. aeruginosa*. Since GM-50 is active on clinical strains, it represents a starting point for identifying and developing new phenolic derivatives acting as QS-inhibitors in *P. aeruginosa* infections.

## Introduction

The misuse of antibiotics has dramatically sped up the spread of multi-drug resistant bacterial pathogens, increasing the occurrence of infections that are challenging to treat (Boucher et al., 2009). Thus, it is not surprising that pharmaceutical companies consider the R&D for new antimicrobial drugs less attractive than other therapeutic areas—i.e., neurodegenerative diseases, cancer, and metabolic diseases. Although developing a novel antibiotic needs significant investments, the economic reward is uncertain because of the unsure commercial life of antimicrobial drugs due to the possible rapid emergence of resistant strains (Luepke et al., 2017). Moreover, the ongoing pandemic of coronavirus disease may have a complex long-term impact on antibacterial resistance (Rawson, 2020). A very recent study reported an odd use of broad-spectrum antimicrobials compared to a low incidence of bacterial infection in hospitalized COVID-19 patients (Lansbury et al., 2020). Thus, the COVID-19 pandemic causes unnecessary antimicrobial use and possibly boosts multidrug-resistant (MDR) bacteria (Rawson et al., 2020).

As the antibacterial effects of antibiotics are associated with selective pressure and the occurrence of resistance, virulence mechanisms have been pointed out as molecular targets for novel drugs since they should hamper the infection process, avoiding effects on bacterial growth (Maura et al., 2016). Quorum Sensing (QS) signaling controls bacterial virulence and provides an ideal target for developing a new class of antibacterial molecules. QS is a cell-to-cell communication system by which bacteria harmonize the expression of virulence factors and tune their behaviors based on bacterial population density and the release/reception of signal molecules (Kalia et al., 2019).

Pseudomonas aeruginosa (PA) has been listed among the MDR nosocomial pathogens that require new therapeutic strategies. The last report of the European Antimicrobial Surveillance pointed out that 31.8% of PA clinical isolates are resistant to at least one of the antibiotic groups under surveillance (EARS-net, 2020). In Italy, it was observed that in 2018 PA reported the highest resistance to piperacillin-tazobactam (23.9%), fluoroquinolones (22.9%), ceftazidime (19.9%), carbapenems (15.8%), and aminoglycosides (13.7%) (Bellino et al., 2018). Moreover, epidemiological studies have revealed that PA rapidly develops resistance to imipenem, tobramycin, ciprofloxacin, and aztreonam, the first-line antibiotics used to treat PA infections (Obritsch et al., 2004; Hill et al., 2006). PA establishes acute or chronic infections by regulating the expression of metabolic pathways and virulence factors, mainly through QS-signalling (Crousilles et al., 2015). In particular, QS in PA is characterized by at least four circuits deeply interconnected: LasI-LasR, RhlI-RhlR, the quinolone-signal-system, and the IQS circuit. The circuits respectively respond to 3-oxo-*N*-[(3*S*)-2-oxooxolan-3-yl]dodecanamide (3OC12-HSL), *N*-[(3*S*)-2-oxooxolan-3-yl]butanamide (C4-HSL), and 2-heptyl-3-hydroxy-1*H*-quinolin-4-one (PQS) to produce 2-(2-hydroxyphenyl)-1,3-thiazole-4-carbaldehyde (IQS). These QS circuits are hierarchically organized, with the Las system at the top of the cascade (Thi et al., 2020). LasI-LasR system includes LasI synthetase that catalyzes the production of 3-oxo-C12-HSL (AHL) as the signal molecule. Once secreted in the extracellular environment, AHL diffuses inside nearby bacterial cells and binds to the LasR receptor. The AHL-LasR complex activates the expression of target genes coding for LasI-synthetase, LasA and LasB elastases, alkaline protease, and genes of the Rhl and PQS QS systems. In the RhlI-RhlR system, the Rhl-synthetase produces the N-butyryl-L-homoserine-lactone, which diffuses in the extracellular space to enter adjacent cells by diffusion and binds the receptor RhlR. The ligand-receptor complex promotes the production of rhamnolipids, LasB elastase, pyocyanin, hydrogen cyanide, and RhlI (Lee and Zhang, 2014). Blocking the quorum sensing signaling may disarm the pathogen by interfering with the coordinated production of its virulence factors such as toxin production, biofilm organization, or making PA more vulnerable to antibiotics. Specifically, more than 300 genes of PA are regulated by QS, that therefore acquires a critical role in *P. aeruginosa* pathogenicity and drug resistance (Girard and Bloemberg, 2008). Among innovative molecules showing QS inhibitory activity, phenolic compounds show interesting properties (Hossain et al., 2017; Langendonk et al., 2021). For example, naringenin, a flavonoid QS inhibitor, competes with AHL-induced LasR activation but only at low *P. aeruginosa* population cellular densities, a situation not representative of common clinical infection (Piepenbrink et al., 2020). Moreover, natural phenolic derivatives such as salicylic acid and trans-cinnamaldehyde possess a QS inhibitory activity only at high or toxic concentrations (Ahmed et al., 2019). Therefore, it is desirable to develop novel phenolic derivatives to identify more effective compounds.

In this study, we screened an in-house library of phenolic derivatives to identify novel QS inhibitors in PA. The tested compounds (either monocyclic or bicyclic derivatives) are derived from our extensive works on cumarins and chromones (Guiotto et al., 1984; Rodighiero et al., 1997; Chilin et al., 2008; Borgatti et al., 2011) for whose synthesis the phenols constitute key intermediates. After *in-vitro* experiments, we corroborated our results in the *G. mellonella* larvae infection model. Since PA clinical isolates differ from the laboratory-adapted strain PAO1 for the variability in the phenotype, we tested the most promising antivirulence molecules on 39 PA strains isolated from the respiratory tract of patients with chronic obstructive pulmonary disease (COPD). Overall, the compound GM-50 exhibited the most significant antivirulence activity, enhancing aztreonam efficacy on PA biofilm formation. We deem that this molecule is a starting point to developing a new pharmacophore of QS inhibitors active in *P. aeruginosa* infections.

## Materials and methods

### Bacterial strains and growth conditions

We used *P. aeruginosa* (CCUG241, Culture Collection University of Gothenburg) (Nakano et al., 2015) (PAO1) cultured in Luria-Bertani (LB) broth. Bioluminescence assays for the detection of the production of AHLs by *P. aeruginosa* were conducted with two different *Vibrio harveyi* strains (Henke and Bassler, 2004). We used *Vibrio harveyi BB120* (HAI-1 positive; AI-2 positive; CAI-1 negative) and *Vibrio harveyi* JAF548. The last one was used as a negative control since the bioluminescence is not controlled by QS systems due to the permanent activation of LuxO, the QS-dependent bioluminescence repressor (Freeman and Bassler, 1999). Both strains were cultured at 30°C in Marine Agar/Broth pH 7.0 (MA/MB, Difco) and experiments were performed in Autoinducer Bioassay medium (AB, Difco) prepared as described (Greenberg and Hastings, 1979).

### Bacterial isolates

Thirty-nine strains of *P. aeruginosa* were isolated from sputum specimens, samples representative of the lower respiratory tract, exanimated, and identified at the Clinical Microbiology Laboratory of Padua University Hospital by MALDI-TOF mass spectrometry. Antibiotic susceptibility was assessed by broth microdilution (BMD) using Sensititre™ Gram Negative Susceptibility Testing Plate (Trek diagnostic system) following Eucast breakpoint 2021.

### Selection of compounds

Based on the recently reported evidence that phenolic (Hossain et al., 2017) and fused phenolic compounds (Langendonk et al., 2021) negatively regulate the QS, we tested our in-house library of phenolic derivatives (81 compounds). The library contained monocyclic phenol derivatives and fused polycyclic phenols (cumarines and chromones). Compounds were initially examined for their ability to interfere with the release of AHLs in *P. aeruginosa* planktonic cultures by bioluminescence assay.

### Bioluminescence assay

To screen the effect of compounds on AHL signaling, PAO1 (10^6^ CFU/ml) was cultured for 24 h (hrs) at 37°C in LB broth supplemented with 100 µM of specified compounds previously dissolved in dimethyl sulfoxide (DMSO; Merck) or with the same volume of DMSO. Simultaneously, *Vibrio harveyi* BB120 was grown in Marine Broth at 30°C for 16 h with shaking. Cultures of *V. harveyi* were collected and 1:5,000 dilutions into fresh AB medium were prepared. Then, 90 μl of diluted *V. harveyi* cultures were mixed with 10 μL of sterile-filtered culture media of PAO1 (treated or not with compounds) containing all the QS molecules released by PAO1, including AHL and AI-2 able to stimulate the *V. harveyi* bioluminescence. The mixtures were seeded into a sterile 96-well white microtiter plate (Costar). Plates were incubated overnight at 30°C and luminescence was quantified with a MultiPlateReader VictorX2 (Perkin Elmer) (Waters et al., 2006). *V. harveyi* bioluminescence was normalized to bacterial growth (OD 620 nm). PAO1 not exposed to any compound was used as positive control and its bioluminescence (normalized to bacterial growth) was arbitrarily set to 100% of the signal.

### AHL analysis

The analysis of QS molecules released by PAO1 is based on previously published procedures (Ortori et al., 2011; Liu et al., 2019) and adopts LC-MS technology using reverse phase chromatography. PA01 (10^6^ CFU/ml) was cultured in LB with and without compounds and incubated at 37°C. Culture supernatants were collected at specified time points (0, 4, 8, 16, 24, 36, and 48 h) and sterilized by filtration. An aliquot was used to quantify the AHL. The instrument comprises an Agilent 1260 LC system combined with a Varian 500MS equipped with Electrospray Ion source (ESI). The method worked with nebulizer pressure at 30 psi, drying gas pressure at 15 psi, drying gas temperature at 290°C capillary voltage 80 V, Rf loading 80%. Column was an Eclipse XDB C18 2,1 × 150 mm (3.5 micron). The gradient started with 60% water, 1% formic acid (A), and 60% ACCN, then in 10 min went to 4% A and remained isocratic up to 12 min (min). Then back to the initial condition at 14 min. The compounds were detected on the basis of their *m/z* value and literature comparison (Ortori et al., 2011; Liu et al., 2019) as C6HSL (*m/z* 200), 3-oxo-C9-HSL (*m/z* 256), *N*-[(3*S*)-2-oxooxolan-3-yl]dodecanamide (m/z 256), 3-oxo-*N*-(2-oxotetrahydro-3-furanyl)dodecanamide (*m/z* 270), 3-oxo-C12-HSL (*m/z* 284), 3-oxo-C12-HSL isomer 1 and 2 (*m/z* 298). Calibration curves were obtained using the reference compound 3-oxo-C12-HSL (Merck) in the concentration range of 0.5–150 μg/ml.

### Growth inhibition assay

Molecules that showed a ≥50% decrease in PAO1-induced bioluminescence in *V. harveyi* BB120 were further evaluated for their potential toxic effect on PAO1. Overnight cultures of PAO1 were collected, centrifuged, and suspended in LB-broth at 10^8^ CFU/ml. Bacteria were dispensed in sterile 96-well microtiter plates at a final concentration of 1 × 10^6^ CFU/100 µL of LB broth/well. PAO1 was cultured with DMSO or in the presence of selected molecules for up to 36 h at 37°C. Bacterial growth was monitored by measuring the optical density at 620 nm at different time points.

### Cytotoxicity assay

To exclude cytotoxic effects on eukaryotic cells, the selected molecules were tested on A549 (CCL-185, ATCC) cell line as a model of the human respiratory tract epithelium. Cells were cultured in DMEM supplemented with 10% FBS and 1% penicillin/streptomycin (all provided by Gibco) and then seeded in 96 well/plates. After 24 h, compounds were added to a final concentration ranging from 0 to 10 mM. Following overnight incubation at 37°C, the culture medium was substituted with fresh one. Following additional 48 h of incubation at 37°C, cells were incubated for 4 h at 37°C with MTT solution (5 mg/ml, Merck). Formazan crystals were solubilized in 100 µL of SDS 10% w/vol, HCl 0.01 N, and the absorbance was recorded 16 h later at 590 nm using a microplate reader (Varioskan Lux Reader, Thermo Fisher Scientific).

### RNA isolation and measurement of gene expression by qRT-PCR

PAO1 was cultured in LB with DMSO or supplemented with the selected molecules for 24 h at 37°C. Then cultures were centrifuged, the medium discarded and total RNA was isolated and purified using the GRS Total RNA Kit—Bacteria (#GK16.0100, GRISP Research Solution) with mechanical and enzymatic cellular disruption, according to the manufacturer’s instructions and as previously described (Berbabè et al., 2021). A treatment with DNase I was used to remove contaminating DNA. The RNA yield and purity were assessed by measuring the ratio of UV absorbance at 260 and 280 nm and only samples in the range of 1.8-2 were used. In preliminary experiments, we observed that expression of most AHL-regulated genes peaked after 24 h of culture (Supplementary Figure S1); thus PAO1 cultures were collected after 24 h of incubation.

The cDNA was generated using a high-capacity cDNA RT kit with an RNAse inhibitor (Applied Biosystems) using a Bio-Rad thermocycler. Quantitative PCR (qRT-PCR) was conducted to determine the levels of transcripts of interest using specific primers indicated in Table 1. The housekeeping gene *proC* was used. The data were analyzed using the 2^ΔΔCt^ method, PAO1 cultures without treatment were used as reference. The qRT-PCRs were conducted using SYBRR green mixture (iScript One-Step RT-PCR kit with SYBR green, Bio-Rad). Annealing temperature and primer sequences and amplicon sizes are reported in Table 1. Samples were assayed in triplicate.

**TABLE 1:** Oligonucleotides sequences and annealing conditions used in the qRT-PCR experiments.

Gene	Primers used for qRT-PCR	Annealing temp (°C)
*proC* (HK)	fw 5′- CAG​GCC​GGG​CAG​TTG​CTG​TC -3′	60
	rv 5′- GGT​CAG​GCG​CGA​GGC​TGT​CT -3′	
*lasI*	fw 5′- GGC​TGG​GAC​GTT​AGT​GTC​AT -3′	60
	rv 5′- AAA​ACC​TGG​GCT​TCA​GGA​GT -3′	
*lasR*	fw 5′- ACG​CTC​AAG​TGG​AAA​ATT​GG -3′	60
	rv 5′-TCG​TAG​TCC​TGG​CTG​TCC​TT -5′	
*rhlI*	fw 5′- AAG​GAC​GTC​TTC​GCC​TAC​CT -3′	60
	rv 5′- GCA​GGC​TGG​ACC​AGA​ATA​TC -3′	
*rhlR*	fw 5′- CAT​CCG​ATG​CTG​ATG​TCC​AAC​C-3′	60
	rv 5′- ATG​ATG​GCG​ATT​TCC​CCG​GAA​C -3′	
*lasB*	fw 5′-GAC​CGA​GAA​TGA​CAA​AGT​GGA​A -3′	60
	rv 5′- GGT​AGG​AGA​CGT​TGT​AGA​CCA​GTT​G -3′	
*rhlA*	fw 5′- TGG​CCG​AAC​ATT​TCA​ACG​T -3′	60
	rv 5′- GAT​TTC​CAC​CTC​GTC​GTC​CTT -3′	
*phzA*	fw 5′- CGA​GGA​TCC​GAA​CCA​CTT​CT -3′	60
	rv 5′- AAC​GGC​TAT​TCC​CAA​TGC​AC -3′	

### Effect of selected molecules on PAO1 biofilm resistance to antibiotic treatment

Selected compounds were tested for their direct or synergistic activity with conventional antibiotics on PAO1 biofilm formation. To better reproduce the growth condition in the lung, we used the Artificial Sputum Medium (ASM) prepared as described by Kirchner (Kirchner et al., 2012). ASM is a culture medium developed to mimic chronic lung colonization by *P. aeruginosa* helpful in evaluating therapeutic procedures and studying antibiotic-resistance mechanisms (Sriramulu et al., 2005). An overnight culture of PAO1 grown in LB at 37°C was diluted in ASM at 10^8^ CFU/ml and 1 ml was seeded into 24 well polystyrene plates and incubated at 37°C with low shaking (75 rpm/min). After 36 h of incubation, the bacterial biofilm was disrupted using 100 μL of cellulase (100 mg/ml, Merck, diluted in 0.05 M citrate buffer [9.6 g/L Citrate in water; pH to 4.6 with NaOH]) and incubated at 37°C with shaking (150 rpm/min) for 1 h. To determine viable cells released from disrupted biofilms, we added 100 μL of 0.02% (v/v in distilled water) resazurin to each well. The plates were incubated at 37°C for 3 h with shaking (150 rpm/min). The non-fluorescent resazurin (blue) is reduced to highly fluorescent resorufin (pink) by dehydrogenase enzymes in metabolically active cells. Thus, the amount of resorufin produced is proportional to the number of viable bacteria. The resorufin formed in the assay was quantified by measuring the relative fluorescence with Multi Plate Reader Victor X2 (Perkin Elmer) (Ex = 560nm, Em = 590 nm). In preliminary experiments, we determined that PAO1 biofilm formation in ASM increased for 24 h and then plateaued until 40 h (Supplementary Figure S2A). Thus, we decided to add treatments (QS inhibitor, 100 µM ± antibiotic, MBIC_50_) after 24 h of biofilm incubation (effect on pre-formed biofilm). The amount of viable bacteria in the biofilm was quantified after additional 16 h (Kirchner et al., 2012). The Minimum Biofilm Inhibitory Concentration (MBIC) of aztreonam was determined by adding different concentrations of the antibiotic to PAO1 seeded in ASM for 24 h and the MBIC_50_ settled at 4 μg/ml (Supplementary Figure S2).

### Virulence factors analysis

Pyocyanin production: The production of pyocyanin was investigated as described by Ugurlu *et al.* (Ugurlu et al., 2016). Overnight cultures of PAO1 or clinical isolates were collected, centrifuged, and resuspended in LB at 10^8^ CFU/ml. Then 10^6^ CFU were incubated at 37°C in LB with DMSO or supplemented with selected compounds. After 24 h, cultures were centrifuged, clear supernatant was collected, and chloroform was added at a 3:5 (v/v) ratio and mixed by inversion. The separated chloroform layer containing pyocyanin was transferred into a new collection tube and acidified with 0.2 M HCl. This reaction led to a pink solution that was aliquoted into a 96-well microtiter plate and optical density at 520 nm was quantified with a microplate reader (Varioskan Lux Reader, Thermo Fisher Scientific).

Elastase B production: Elastolytic activity of PAO1 and clinical isolates was assessed following the method described by Parasuraman *et al.* (Parasuraman et al., 2020). Samples were prepared as described above and cell-free supernatants were added (1:9 v/v ratio) to Elastin Congo Red buffer (ECR buffer: 100 mM Tris, 1 mM CaCl_2_, pH 7,5) containing 20 mg of Elastin Congo Red (Merck). The mixtures were incubated at 37°C for 3 h with shaking (150 rpm/min). After incubation, tubes were centrifuged (10,000 rpm for 5 min) to remove the insoluble material. One hundred microliters of the supernatants were transferred in a 96 well microplate and optical density at 540 nm was quantified with a microplate reader (Varioskan Lux Reader, Thermo Fisher Scientific).

Swarming motility: *P. aeruginosa* culture samples were prepared as described in pyocyanin quantification. After overnight incubation, 10 µL of the culture previously diluted 1:10 was seeded on Agar plates containing medium formulated with 1% tryptone, 0.5% NaCl, 0.5% agar, and 0.5% filter sterilized di D (+)-Glucose (Merck). The plates were incubated at 37°C in the upright position for 36 h and then photographed. The distance movement was calculated using the freely available software ImageJ. Plates inoculated with not-treated PAO1 were used as the positive control (Berbabè et al., 2021).

Rhamnolipids quantification: rhamnolipids quantification was performed as described before with some modifications (Rasamiravaka et al., 2016). PAO1 cultures, treated with GM-50 or DMSO, were grown under shaking (175 rpm) at 37°C for 24 h in LB-MOPS medium (Difco). At the end of incubation, cultures were centrifuged (3,200 × g; 24°C; 5 min). Ten mL of supernatant were filtered to remove residual cells and pH was adjusted to 2/3 using 1N HCl. Then ethyl acetate was added at a 1:1 (v/v) ratio and vigorously vortexed for 20 s. The upper phase was evaporated into a new tube in a speed vac centrifugal evaporator. Dry rhamnolipid extracts were then dissolved in 2 ml chloroform and mixed with 200 μL of methylene blue solution (Merck). Tubes were vigorously mixed and after phase separation (15 min) the lower phase (chloroform) was transferred to a new tube and HCL 0.2N was added at a 1:2 (v/v) ratio. Samples were vortexed and left at room temperature for 10 min. Finally, the upper acid phase was transferred to a 96-well microplate and the absorbance was measured at 638 nm.

### Cytokine assays

To assess the ability of PAO1 to induce an inflammatory phenotype in epithelial cells, we evaluated the release of interleukin (IL)-8 and IL-6 in human alveolar epithelial cells A549. PAO1 was cultured in LB medium supplemented with GM-50 at 100 µM or equal volume of DMSO. After 24 h, bacteria were collected, centrifuged, and added to A549 monolayers at a MOI of 1:20 (eukaryotic cells: bacteria). Bacteria and cells were incubated at 37°C and 5% CO_2_. After 2 h, the culture medium was removed, cells were washed with PBS 1X, and monolayers were incubated with DMEM containing 200 μg/ml gentamycin sulfate (Merck) to kill residual bacteria. After 24 h of incubation at 37°C, the supernatants were collected and centrifuged at 13,000 rpm for 6 min. Then, clear supernatants were stored in aliquots at −80°C until cytokine quantification by ELISA. The levels of IL-8 and IL-6 were measured using human IL-8 and IL-6 ELISA kits (ImmunoTools) according to the manufacturer’s protocol and as described elsewhere (Carta et al., 2018). Each tested sample was derived from three independent experiments run in triplicate.

### Quantification of associated and internalized bacteria

To determine the effect of selected compounds on PAO1 adhesion to or internalization in epithelial cells, we followed procedures described by Hawdon *et. al* (Hawdon et al., 2010) with some modifications. A549 cells (10^5^/well) were seeded on 24 well plates and cultured for 2 days until they reached approximately 90% confluence. PAO1, cultured for 24 h in LB medium supplemented with DMSO or GM-50 at 100 µM, were collected, centrifuged, and added to A549 monolayers at an MOI of 1:20 (eukaryotic cells: bacteria). Bacteria and cells were incubated for 2 h at 37°C and 5% CO_2_; after that, the culture medium was removed, and cells were washed three times with sterile PBS to remove non-adhering bacteria.

To quantify bacteria associated with epithelial cells, we added 300 µL of 0.05% trypsin-EDTA. Following incubation for 5 min at 37°C, cells were collected and lysed (5 min at 37°C) by adding 700 μL of 0.1% Triton X-100. Lysates were properly diluted, seeded into LB agar plates, and incubated at 37°C for 24 h. Bacterial colonies were counted to enumerate bacteria associated with A549 monolayers. The results represented the amount of both internalized and adherent bacteria (CFU/cell). To specifically enumerate the internalized bacteria, after 2 h of infection, monolayers were washed three times with PBS. Then, DMEM supplemented with 10% FBS, 1% penicillin/streptomycin (Gibco), and 200 μg/ml gentamicin sulfate was added to each well to wipe out extracellular bacteria. After 90 min, the culture medium was removed, and cells were washed three times and lysed as described above. After proper dilutions, samples were seeded in LB agar plates and incubated at 37°C and the colonies enumerated after 24 h represent the number of internalized bacteria. All experiments were performed three times with triplicate determinations.

### *In vivo* assays

To investigate the ability of selected compounds to control *P. aeruginosa* infection, we used *Galleria mellonella* larvae, an inexpensive invertebrate model with no ethical constraints. These larvae possess innate immune responses and are ideal for rapid *in vivo* studies (Tsai et al., 2016). Larvae (∼500 mg) were inoculated with 10 CFU of PAO1. Two hrs later, larvae were injected with 100 μM GM-50 or vehicle (PBS +0.05% DMSO). Compound GM-32 resulting as inactive at the first screening was used as a negative control. Survival was recorded for up to 40 h in treated and not-treated invertebrates. All experiments were conducted three times with 30–50 determinations for each condition.

### Computational methodology

All the computational methodologies were carried out on a 32 Core AMD Ryzen 9 3905X, 3.5 GHz Linux Workstation (O.S. Ubuntu 20.04) equipped with GPU (Nvidia Quadro RTX 4000, 8 GB).

Protein structures. The structure of LasR was retrieved from the Protein Data Bank (PDB-ID: 6d6a, chain A) (O’Reilly et al., 2018), whereas the structures of RhlR, LasI and RhlI were obtained from the AlphaFold Protein Structure Database (IDs: A0A411HE41, P33883, A0A411F6V2, respectively) (Jumper et al., 2021; Viradi et al., 2022).

Molecular docking simulations. The three-dimensional structures (”.mol2” format with explicit hydrogenation at pH = 7.2) of the compounds GM-50, C4HSL, and 3-oxo-C12-HSL were prepared with OpenBabel ver. 3.1.1 starting from the SMILES code (O’Boyle et al., 2011) and then converted into the appropriate “.pdbqt” files using AutoDock (AD) ver. 4.2 (Morris et al., 2009). All the protein structures were aligned to the LasR structure and the appropriate “.pdbqt” files were prepared using AutoDock (AD) ver. 4.2. The following binding complex were simulated: GM-50/LasR, GM-50/RhlR, GM-50/LasI, GM-50/RhlI, 3O-C12-HSL/LasR, C4-HLS/RhlR. All the docking simulations were conducted in a box (centre: *x* = 57.502, *y* = 15.631, *z* = 6.591) with grid spacing = 0.375 Å and 40 × 40 × 40 points. The docking poses were generated using the Lemarkian genetic algorithm (Morris et al., 1998) with the following parameters: population size = 250; number of evaluations: 2,500,000; maximum number of generations = 27,000; mutation rate = 0.02; crossover rate = 0.8. For each simulated pair compound/protein, we found that the lowest energy cluster contained the lowest energy pose (that was, in turn, analyzed in-depth to identify the compound/protein interactions) and was also the most populated cluster. The selected binding poses were used as starting structures for molecular dynamics simulations.

Molecular dynamics simulations. All the molecular dynamics simulations were conducted with the Gromacs ver. 2021.1 software (Van der Spoel et al., 2005; Pronk et al., 2013), using the CHARMM36 forcefield (Huang and MacKerell, 2013) for both the proteins and the ligand. The parameters files required for the ligands were obtained through the CGenFF website (Vanommeslaeghe and MacKerell, 2012). The long-range electrostatic interactions were modeled with the PME method. The short-range Coulomb and Van der Waals interactions were subjected to a cut-off of 1.2 nm. The LINCS algorithm was used to constrain the bonds involving hydrogen atoms. Each complex protein/ligand was centered in a cubic box, solvated (water model: TIP3P), and neutralized with Na^+^ and Cl^−^ ions (final concentration: 0.15 M). The structures were then equilibrated in the NVT (200 ps, T = 300°K, v-rescale thermostat, dt = 0.001 fs) and the NPT (200 ps, T = 300°K, *p* = 1 bar, Berendsen barostat, dt = 0.002 fs) ensembles. Production runs were conducted for 50 ns using the Parrinello-Rahman barostat (Parrinello and Rahman, 1981). The root-mean squared deviations and the interaction energies were obtained through the “gmx rms” and “gmx energy” routines.

### Data management and statistical analysis

Clinical isolates were collected at the Clinical Microbiology Laboratory of Padua University Hospital and identified using randomly generated numerical ID. Data relative to patients were kept in a separate database following applicable laws and guidelines on privacy management. Personal data of patients were not shared among the participants of the study.

All reported experiments were conducted at least three times with triplicate determinations for each condition. Data were analyzed by two-way analysis of variance (ANOVA) followed by Bonferroni multiple comparison post hoc test using GraphPad Prism (version 8.0). A *p*-value of 0.05 or less will be considered statistically significant. Cartoons in Figures 4H, 5E, 8 were realized using Microsoft PowerPoint® 2010 and Microsoft Paint®.

## Results

### Bioluminescence assay to identify AHL quorum sensing inhibitor candidates

To screen our library of 81 compounds for interference with the AHL-mediated quorum sensing system of PAO1, we performed a bioluminescence assay using the reporter strain *Vibrio harveyi* BB120, in which bioluminescence emission proportionally increases in response to AHL (Supplementary Figure S1). To identify when AHL reaches its highest level in a PAO1 culture, we collected the PAO1 culture medium at different time points and determined the amount of AHL by adding 10 µL of PAO1 supernatants to *V. harveyi* cultures. Sixteen hrs later, we quantified *V. harveyi*-produced luminescence using Varioskan LUX Multimode Microplate Reader. Bioluminescence of *Vibrio harveyi* BB120 exposed to PAO1 culture supernatants was barely detectable up to 6 h, significantly increased at 16 h, and peaked at 24 h of incubation. No further increase was observed at longer incubation times (Supplementary Figure S1). Moreover, liquid chromatography plus mass-spectrometry analysis of PAO1 culture media confirmed that AHL reached the highest level after 24 h of incubation (Supplementary Figure S1). Indeed, a direct correlation was observed between AHL levels in the PAO1 culture medium and *V. harveyi*-produced luminescence (Supplementary Figure S1). Therefore, the following tests were carried on by treating PAO1 cultures with the specific synthetic small molecules for 24 h and detecting the presence of AHLs using *V. harveyi* BB120. We arbitrarily assigned a 100% luminescence value to the signal produced by *V. harveyi* following incubation with PAO1 + vehicle (DMSO 0.05%). Data relative to the screened 81 compounds are reported in Supplementary Figure S2. Sixty-one molecules were considered ineffective (i.e., they caused an increase in bioluminescence emission or a reduced luminescence emission by less than 20% compared to PAO1 treated with DMSO); twelve molecules reduced the bioluminescence emission only by 21–49% and were abandoned since considered weakly active. Nine molecules reduced AHL-mediated bioluminescence in *V. harveyi* BB120 by more than 50% (Supplementary Table S1). The nine molecules showing significant inhibitory activity were subjected to a second screening assay, evaluating their effect on the transcription levels of AHL-regulated genes by molecular analysis. JAF548-related luminescence did not increase after the addition of PAO1 supernatant (data not shown).

### Inhibition of transcription of *las-* and *rhl-*regulated genes in the presence of selected compounds

**FIGURE 1:**
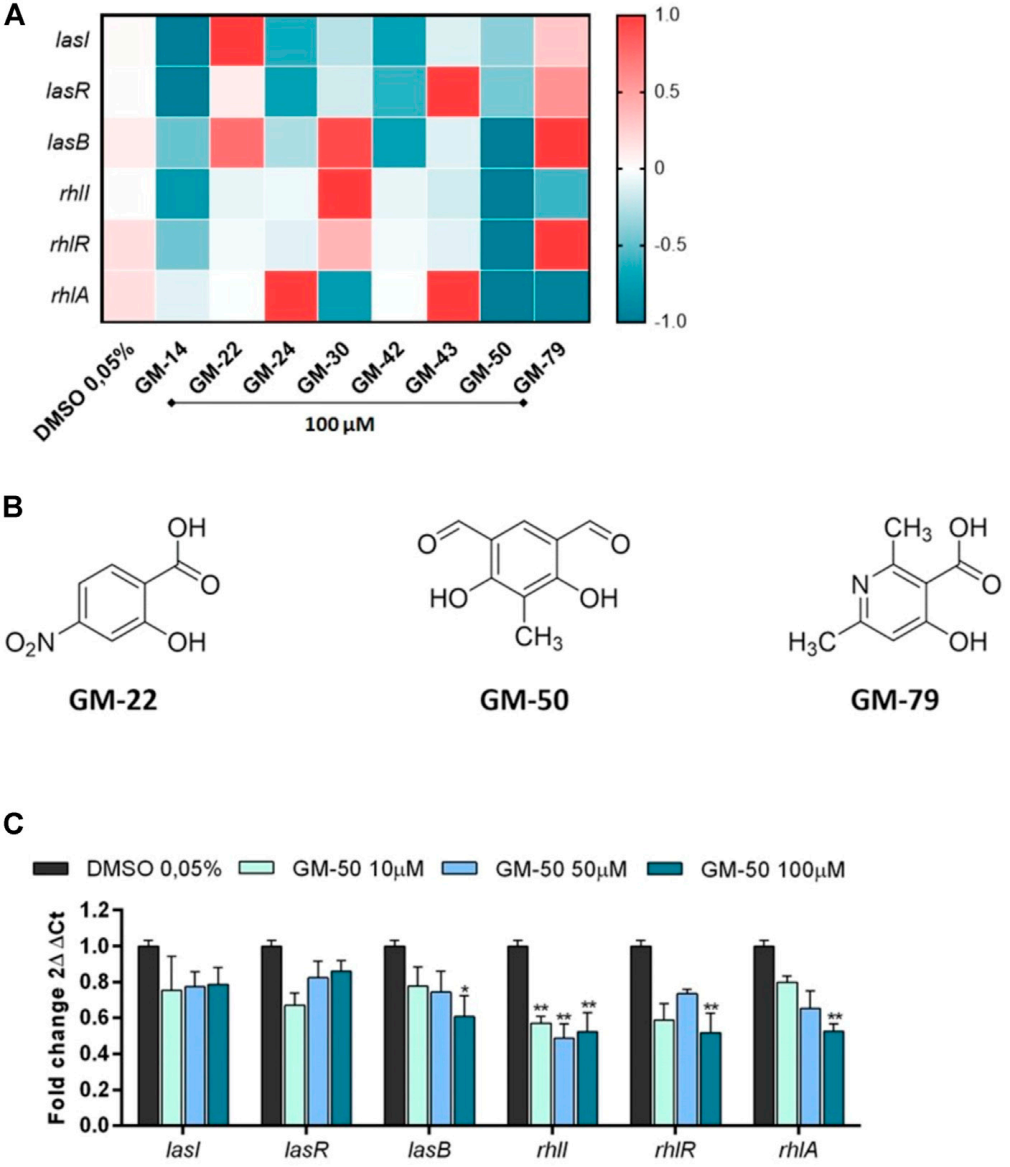
Effect of compounds selected by bioluminescence assay on the expression of AHL-sensitive genes. **(A)** Heatmap illustrating differential gene expression over vehicle-treated controls. PAO1 (10^6^ CFU/ml) cultures were treated with 100 µM of compounds GM-14, GM-22, GM-24, GM-30, GM-42, GM-43, GM-50, GM-79 or with vehicle (DMSO 0.05%) for 24 h at 37°C. Transcript levels of AHL-responsive genes *lasR/lasI/lasB* and *rhlR/rhlI/rhlA,* were assessed by qRT-PCR. Expression values for each gene (rows) are normalized across all samples (columns) by proC expression. Positive correlations are marked in red and negative ones in blue (color scale at top). **(B)** Chemical structures of GM-22, GM-50, and GM-79. **(C)** PAO1 (10^6^ CFU/ml) were cultured in LB with GM-50 (10, 50, and 100 μM). After 24 h, *LasR/lasI/lasB* and *rhlR/rhlI/rhlA* mRNA transcript levels were determined by qRT-PCR. Results are reported as 2^ΔΔCt^ of relative gene expression (*n* = 3). *p < 0.05, **p < 0.01 vs. PAO1 with DMSO 0.05%.

Since the antivirulence activity of AHL-QS inhibitors correlates with the reduced expression of las- and rhl-regulated genes, we determined the ability of the selected 9 phenolic derivatives to interfere with AHL-induced expression of specific genes. Thus, using qRT-PCR we quantified the mRNA transcripts of lasI and rhlI (genes involved in the synthesis of AHLs), lasR and rhlR (genes coding for AHLs receptors), and lasB and rhlA (genes coding for elastase B and rhamnolipids, respectively) in PAO1 cultured for 24 h in the presence of the selected small molecules. Molecular analysis revealed that 2 out of 8 compounds (GM-22 and GM-79; Figures 1A,B) stimulated the expression of AHL-dependent QS signaling systems. GM-50 showed the highest inhibitory activity (Figures 1A,C). GM-50 was the most potent compound in reducing the expression of rhlR/I/A and lasB and we observed a moderate effect on LasR/I (Figures 1A,B). Afterward, we performed a dose-response experiment to identify the lowest effective dose. We found that GM-50 is active on the Las pathway only at 100 μM, whereas it maintains the inhibitory activity on the Rhl pathway also at 10 µM (Figure 1C).

### Compound GM50 does not interfere with bacteria viability and is not toxic in eukaryotic cells

**FIGURE 2:**
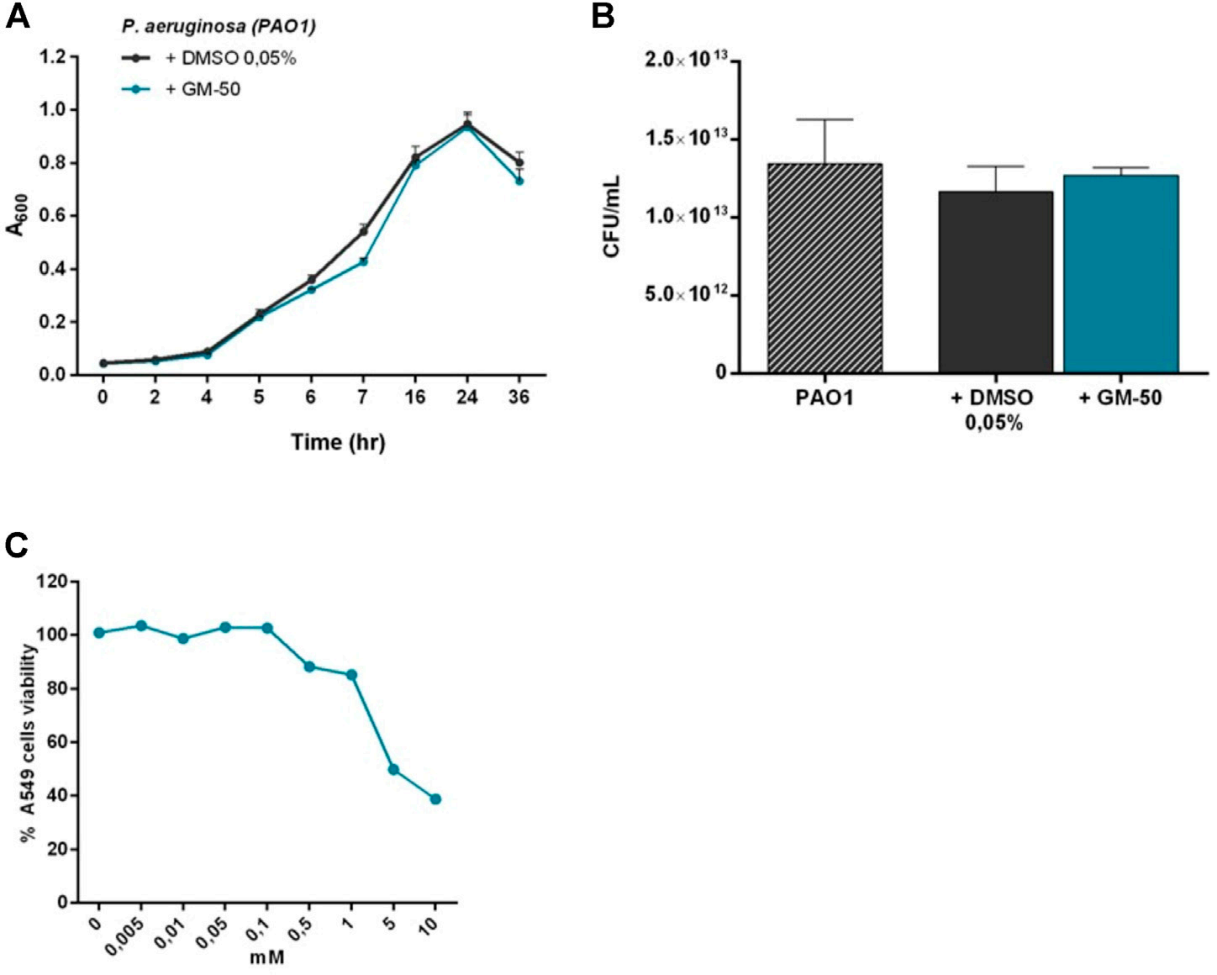
Effect of GM-50 on prokaryotic and eukaryotic cells growth and viability. PAO1 (10^6^ CFU/ml) cultures were treated with 100 µM of GM-50 or vehicle. **(A)** Bacterial growth was monitored for 36 h by measuring optical density (OD = 620 nm). **(B)** Bacterial viability was evaluated by seeding proper culture dilutions in LB agar and enumerating colonies (CFU) after 16 h (*n* = 3) **(C)** Human respiratory tract epithelial cells A549 were cultured with GM-50 at concentrations ranging from 0 to 10 mM. Cell viability was assessed by MTT (3- (4,5-dimethylthiazol-2-yL)-2,5- diphenyltetrazolium bromide) assay. Data (*n* = 3) are reported as percent cell viability calculated over the control (vehicle).

To exclude that GM-50 reduced AHL production because of direct toxicity on bacteria we cultured PAO1 for 24 h in 96 well in the presence of DMSO 0.05% (vehicle) or 100 µM of GM-50. We monitored optical density to measure the microbial growth curve for 36 h. As reported in Figure 2A, GM-50 had no significant effects on bacterial growth at the tested concentration. Moreover, the colony count assay was not affected by GM-50 (Figure 2B).

To investigate GM-50 toxicity, we exposed A549 human lung carcinoma epithelial cell line to the compound at concentrations ranging from 0 to 10 mM for 24 h. Cell viability was measured by MTT assay. GM-50 showed minimal cytotoxic activity at concentrations higher than 200 μM, greater than the one effective on AHL-dependent QS signaling systems (Figure 2C). Overall, GM-50 showed no direct toxicity on prokaryotic and eukaryotic cells.

### GM-50 inhibits AHL production

**FIGURE 3:**
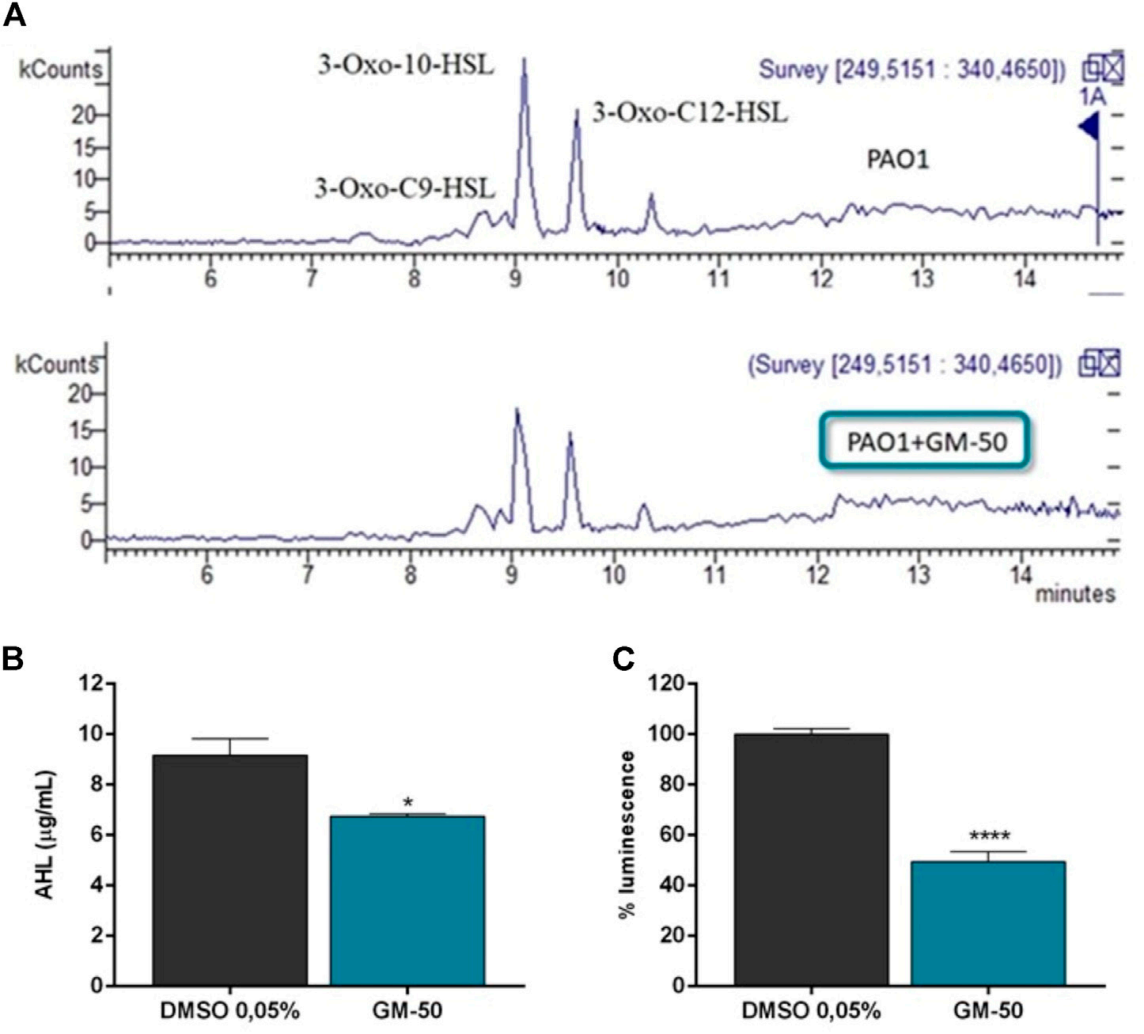
Effect of GM-50 on AHL production. PAO1 (10^6^ CFU/ml) were cultured in LB with GM-50 (100 µM) or DMSO 0.05% (vehicle) for 24 h at 37°C. After incubation, supernatants were used to quantify AHL production adopting LC-MS technology using both reverse phase chromatography and luminescence assay. **(A)** LC-MS chromatogram of collected supernatants. C6HSL (*m/z* 200), 3-Oxo-C9-HSL (*m/z* 256), N-decanoyl-homoserine lactone (m/z 256), 3-Oxo-10-HSL N-dodecanoyl-homoserine lactone (*m/z* 270), 3-oxo-C12-HSL (*m/z* 284), 3-Oxo-C12-HSL isomer 1 and 2 (*m/z* 298) were detected. **(B)** Total quantification of AHL molecules is reported as µg/mL. **(C)** Supernatants were added to *Vibrio harveyi* BB120. Luminescence was quantified after 24 h. Results obtained with PAO1 cultured with DMSO were arbitrarily assigned 100% AHL production. *p < 0.05, **p < 0.01 vs*.* PAO1 with DMSO 0.05%.

The LC-MS measurements allowed us to observe that the treatment with compounds GM-50 inhibits AHL production (Figure 3). As evidenced in the representative chromatograms in Figure 3A, cultures of PAO1 treated with GM-50 showed a reduction in the intensity of the peaks ascribable to the produced AHLs (Figure 3B). This result correlates with the bioluminescence assay results (Figure 3C) and with the ability of the compound to inhibit the AHL-regulated genes (Figure 1C).

### Compound GM-50 strongly reduces the production of virulence factors regulated by AHL-signalling and its gene expression

**FIGURE 4:**
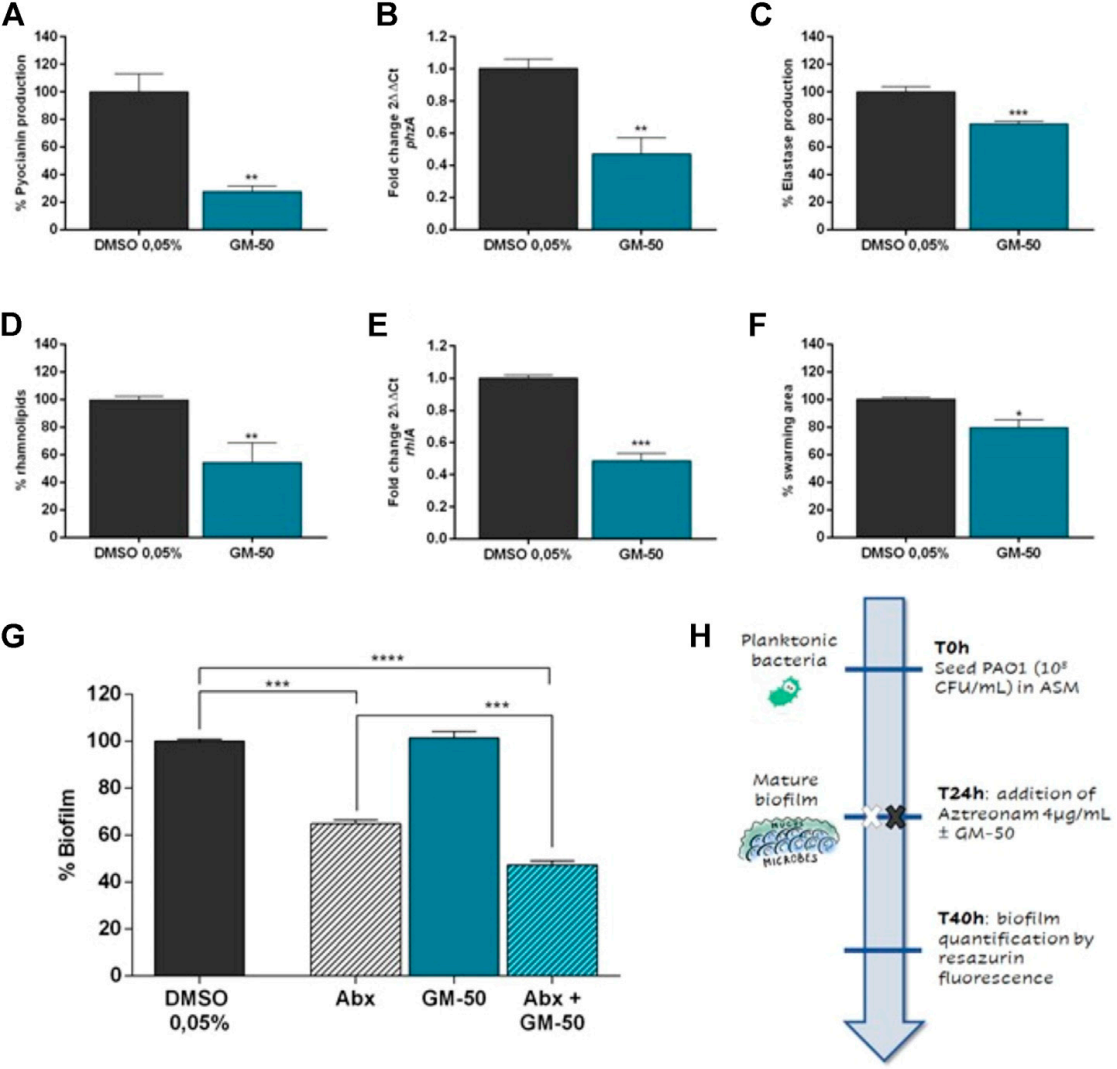
Effect of GM-50 on PA01 virulence factors expression. PAO1 (10^6^ CFU/ml) was cultured in LB with GM-50 (100 µM) or DMSO 0.05% (vehicle) for 24 h at 37°C. After incubation, supernatants were used to quantify **(A)** pyocyanin production by chloroform-HCl method, **(C)** elastase production by Elastin-Congo red assay, **(D)** rhamnolipids release by methylene blue staining. **(B,E)** Transcript levels of *phzA* and *rhlA*, genes involved in pyocyanin and rhamnolipids production respectively, were assessed by qRT-PCR. **(F)** To evaluate swarming, PAO1 cultures were inoculated on the center of LB-agar plates (0.5% agar) and incubated at 37°C. We then evaluated the distance from the center. PAO1 + DMSO 0.05% was used as positive control and was arbitrarily set at 100%. **(G)** PAO1 (10^6^ CFU/ml) was cultured in ASM for 24 h at 37°C with moderate agitation (75 rpm) to form biofilm. PAO1 mature biofilm was treated with aztreonam (Abx, 4 μg/ml), GM-50 (100 µM), or aztreonam (Abx, antibiotic) and GM-50 for 16 h. Residual biofilm was then evaluated by measuring the relative fluorescence units (RFU) using a fluorimeter (Ex = 530–570 nm, Em = 590–620 nm). Data are reported as the percentage of biofilm calculated by setting DMSO as 100%. Data are represented as mean ± SEM, *n* = 3 experiments. *p < 0.05, **p < 0.01, ***p < 0.001, ****p < 0.0001 vs. PAO1 with DMSO 0.05%. **(H)** Schematic representation of the experimental design for biofilm evaluation reported in **(G)**.

AHL-dependent QS signaling systems modulate the expression of a large group of genes regulating virulence in *P. aeruginosa*, such as biofilm formation, swarming motility, pyocyanin synthesis, and elastase B and rhamnolipids production. To verify whether GM-50 inhibited elastase B, pyocyanin, and rhamnolipids production, we compared the production of these toxins in vehicle-treated PAO1 cultures to PAO1 cultures exposed to the compound. After 24 h of incubation, GM-50 significantly reduced the production of virulence factors in PAO1: it was active in reducing pyocyanin (approximately by 70%), rhamnolipid production (by 40%), and elastase B release (by 20%) (Figures 4A,C,D). Notably, GM-50 inhibited the expression of *rhlA* and *phzA*, genes under the QS control and involved in rhamnolipids and pyocyanin synthesis (Figures 4B,E). To evaluate the ability of the compound to perturb swarming motility, PAO1 cultures were first cultured for 24 h in the presence or absence of the compound. They were then seeded in the centre of the agar plates to evaluate bacterial movements. As shown in Figure 4F, GM-50 reduced by 20% PAO1 swarming.

### GM-50 potentiates aztreonam activity in mature PAO1 biofilm

To evaluate the impact of GM-50 on biofilm disruption, we treated PAO1 mature biofilm (24 h of growth with slow agitation). Since previous studies reported that disrupting QS signaling might enhance sensitivity to traditional antibiotics, we decided to test the compounds in combination with Aztreonam, an antibiotic recommended in treating chronic *P. aeruginosa* infections (Brackman et al., 2011). PAO1 incubated with the medium containing DMSO 0.05% was used as a negative control and assigned 100% biofilm formation. We previously identified at 4 μg/ml the aztreonam MBIC_50_ (Supplementary Figure S3). Compared to the negative control, Aztreonam alone reduced biofilm by 40% (Abx, Figure 4G). GM-50 (100 µM) was ineffective on pre-formed biofilm when used alone (Figure 4G). However, the combination of GM-50 with aztreonam at MBIC_50_ (see the experimental design in Figure 4H) reduced by 60% the mature biofilm. Thus, the addition of compound GM-50 significantly enhanced aztreonam activity on biofilm disruption.

### Compound GM-50 reduces PAO1-mediated epithelial cell activation

**FIGURE 5:**
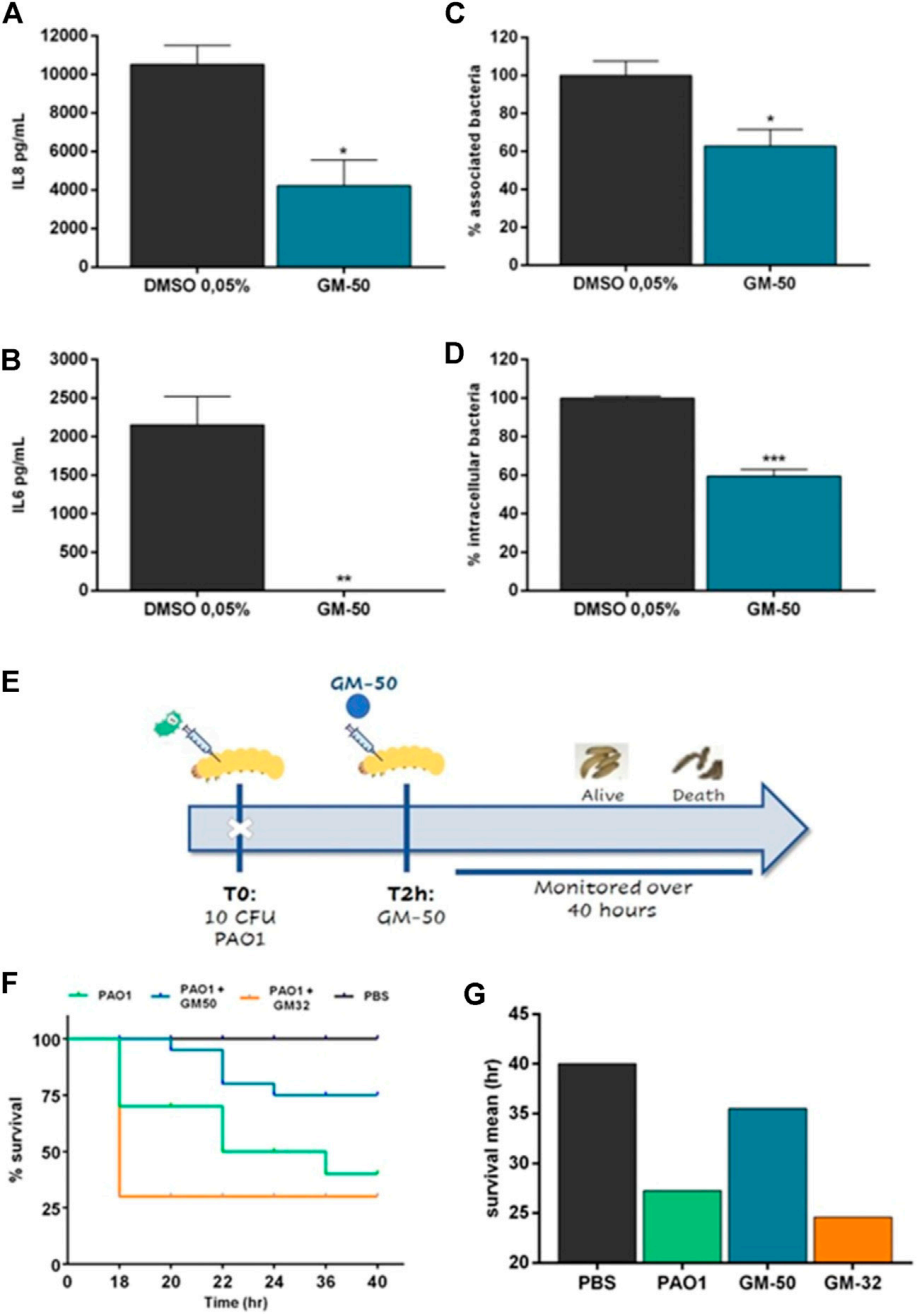
Effect of GM-50 on PA01-epithelial cells interaction and PAO1 *G. mellonella in-vivo* infection model. PAO1 (10^6^ CFU/ml) was cultured in LB with GM-50 (100 µM) or DMSO 0.05% (vehicle) for 24 h at 37°C and used to infect adherent A549 cells monolayers (MOI 1:20) for 2 h **(A,B)** Cells were washed, and cultured for 24 h in fresh DMEM containing gentamycin sulfate (200 μg/ml). IL-8 and IL-6 were quantified by ELISA in conditioned media. **(C)** Cells were washed, collected, lysed, seeded on LB-agar plates, and incubated overnight at 37°C to enumerate total bacteria associated with A549 monolayers (internalized and adherent) expressed as CFU/cell. **(D)** Cells were washed and incubated in media containing gentamycin sulfate. After 2 h cells were washed, collected, lysed, and seeded on LB-agar plates to enumerate internalized bacteria, expressed as CFU/cell. Data are represented as mean ± SEM, *n* = 3 experiments. *p < 0.05, **p < 0.01, ***p < 0.001, ****p < 0.0001 vs. PAO1 with DMSO 0.05%. **(E)** Schematic representation of the experimental design. *G. mellonella* larvae were infected with 10 CFU of PAO1. Two hrs later, larvae were injected into the right proleg with specified compounds and monitored for 40 h. **(F)** Kaplan-Meier survival curve of infected *G. mellonella* receiving no treatment, treatment with GM-50 or GM-32, a molecule devoid of activity on AHL-dependent QS (see Supplementary Material). *p < 0.05 vs. PAO1. **(G)** Average hrs of larvae survival post-infection.

Pulmonary infections caused by *P. aeruginosa* are commonly associated with the activation of intracellular signals resulting in the release of cytokines and chemokines. In particular, IL-8 plays a crucial role in recruiting inflammatory cells enhancing neutrophil-mediated lung injury (Sar et al., 1999). A549 cells infected with PAO1 at a MOI of 1:20 released significant amounts of IL-8 and IL-6 (Figures 5A,B). Incubation with GM-50 significantly reduced PAO1-induced IL-8 and IL-6 secretion (Figures 5A,B).

Bacterial adhesion to airway epithelial cells and internalization are critical steps to colonize and damage the airway mucosa. In *P. aeruginosa*, they are both regulated by QS signaling (Strateva and Mitov, 2011). Therefore, we evaluated the effect of the phenolic derivative GM-50 on PAO1 adhesion and internalization in A549. As reported in Figures 5C,D, GM-50 reduced *P. aeruginosa* adhesion by almost 50% and reduced the internalization of PAO1 in airway epithelial cells by 45%.

### Compound GM-50 decreases PAO1-induced mortality in G. mellonella larvae

We evaluated the *in vivo* effect of treatment with GM-50 in the *G. mellonella* model system (see the experimental design in Figure 5E). The QS inhibitor was not toxic for *G. mellonella* at the concentrations used (data not shown). Following injection of 10 CFU of PAO1, a significant decrease in survival of *G. mellonella* at 40 h was observed, and mean survival time was reported at 27 h compared to PBS injected larvae (Figures 5F,G). Conversely, ∼75% of *G. mellonella* larvae challenged with PAO1 and treated with GM-50 survived 36 h after infection. On the contrary, treatment with the negative control GM-32, a molecule devoid of activity on AHL-dependent QS signaling (Supplementary Figure S2), had no significant effect on PAO1-induced *G. mellonella* larvae mortality and mean survival time (Figure 5G). Overall, our results demonstrate that GM-50 was able to protect *G. mellonella* larvae from *P. aeruginosa* PAO1 infection.

### GM-50 inhibits the production of virulence factors in clinical isolates

**FIGURE 6:**
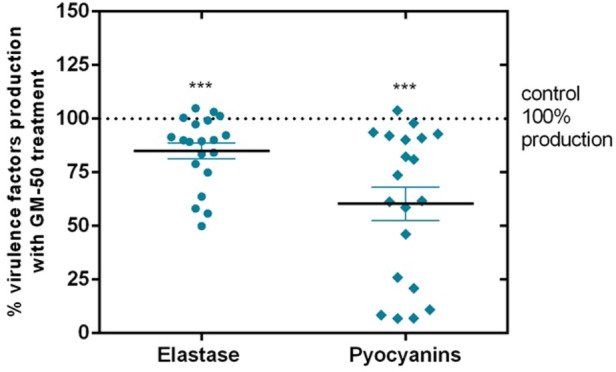
Effect of GM-50 on virulence factors expression in *P. aeruginosa* clinical isolates. *P. aeruginosa* respiratory tract isolates were cultured (10^6^ CFU/ml) in LB with GM-50 (100 µM) or DMSO 0.05% (vehicle) for 24 h at 37°C. After incubation, supernatants were used to quantify elastase release by Elastin-Congo red assay or pyocyanin production by the chloroform-HCl method. Pyocyanin and elastase obtained with strains cultured with DMSO 0.05% were arbitrarily assigned 100% production and were used as control. Data are represented as mean ± SEM, *n* = 3 experiments for each clinical isolate. ***p < 0.001 vs*.* control.

Since laboratory strain PAO1 significantly differs from field strains, we collected 20 *P. aeruginosa* isolates from the sputum of patients with respiratory tract infections. We assessed the anti-virulence activity of GM-50 on these clinical isolates. For each isolate, the values of the virulence factors obtained by incubating the strain in LB containing only DMSO 0.05% were set as the 100% of the activity. As expected, the compound GM-50 was not active on all strains probably due to genetic modifications acquired by clinical isolates. However, as reported in Figure 6, GM-50 significantly reduced elastase and pyocyanin production in almost all isolates. Remarkably, GM-50 is strongly active in pyocyanin inhibition, a virulence factor mainly regulated by rhl-QS pathway (Dekimpe and Déziel, 2009). Moreover, these results are in accordance with qRT-PCR pointing out the rhl-pathway as the GM-50 target.

### GM-50 docking and molecular dynamics simulations

To dissect the mechanisms of action of GM-50 on multiple QS systems of *P. aeruginosa,* we performed molecular docking simulations focusing on RhlR, RhlI, LasR and LasI, four proteins involved in the two QS pathways regulating the production of the virulence factors described above. While in the case of LasR different three-dimensional structures were present in the Protein Data Bank repository, the structures of RhlR, LasI, and RhlI were derived from the AlphaFold repository. AlphaFold is a well-recognized recently developed artificial intelligence program that is able to predict the 3D structures of (potentially) any protein (Jumper J et al., 2021; Varaldi M et al., 2022). For comparison purposes, C4HSL and 3-oxo-C12-HSL, the physiological activator of RhlR and LasR respectively were also docked in the receptor. The two AHLs were not docked in LasI and RhlI since it is conceivable that both C4HSL and 3-oxo-C12-HSL are readily released from the synthases upon production.

**FIGURE 7:**
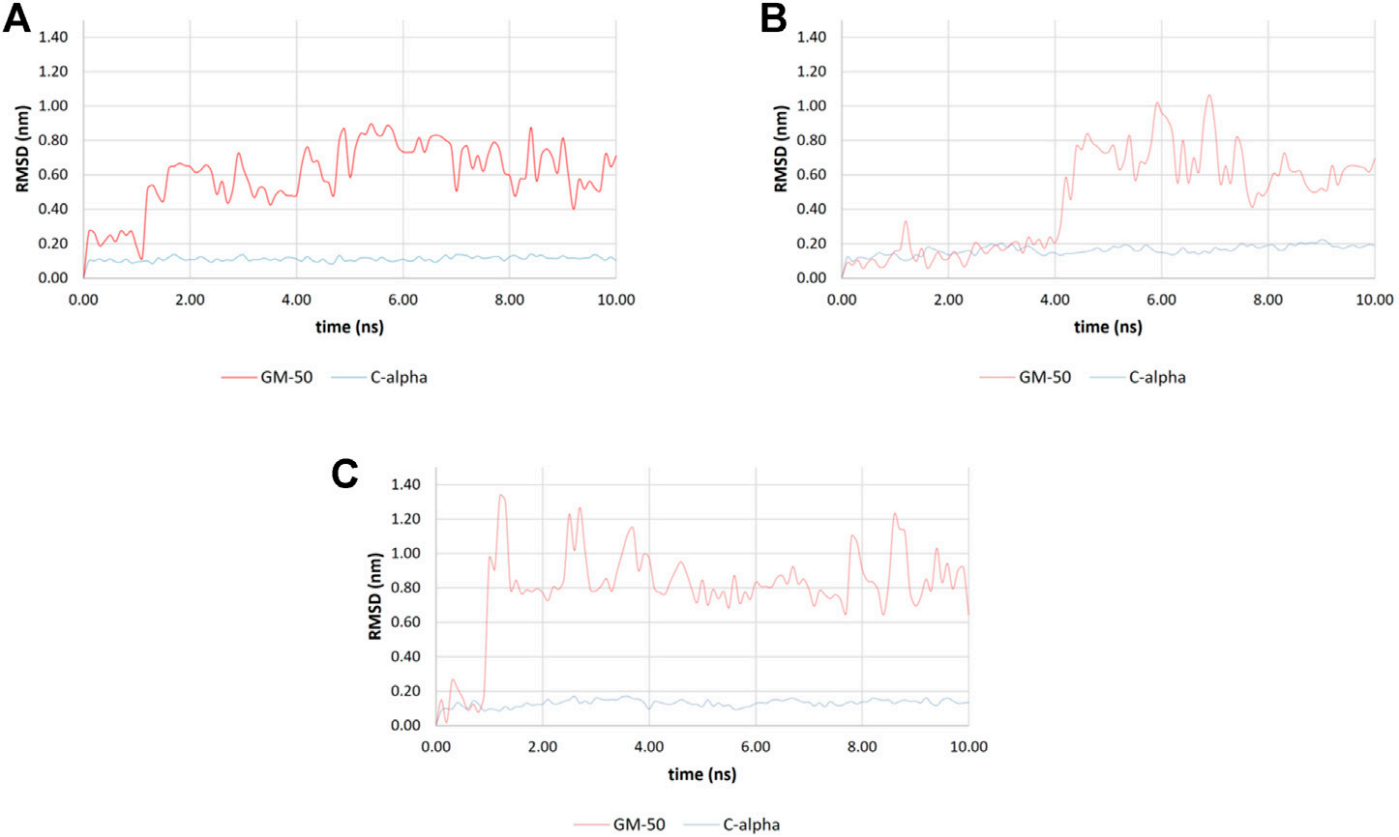
Molecular docking simulations. Root mean square deviation (RMSD) values were obtained during the first 10 ns of molecular dynamics simulations. **(A)** RMSD values for the LasR C-alpha atoms (blue line) and GM-50 (displacement of ligand heavy atoms with respect to protein C-alpha atoms; red line). **(B)** RMSD values for the RhlI C-alpha atoms (blue line) and GM-50 (displacement of ligand heavy atoms with respect to protein C-alpha atoms; red line). **(C)** RMSD values for the LasI C-alpha atoms (blue line) and GM-50 (displacement of ligand heavy atoms with respect to protein C-alpha atoms; red line). In all the cases, while the proteins resulted stable during the simulations, the ligand was readily displaced from the binding site (RMSD >0.6 nm within 10 ns).

The predicted complexes were then submitted to molecular dynamics (MDs) simulations, to verify the stability (and thus the reliability) of the docked structures. The complexes GM-50/LasR, GM-50/RhlI, and GM-50/LasI resulted unstable; the compound was readily released from the predicted binding sites and the simulations were stopped after 10 ns (Figures 7A,B,C, respectively), suggesting that these proteins are not targeted by the compound.

**FIGURE 8:**
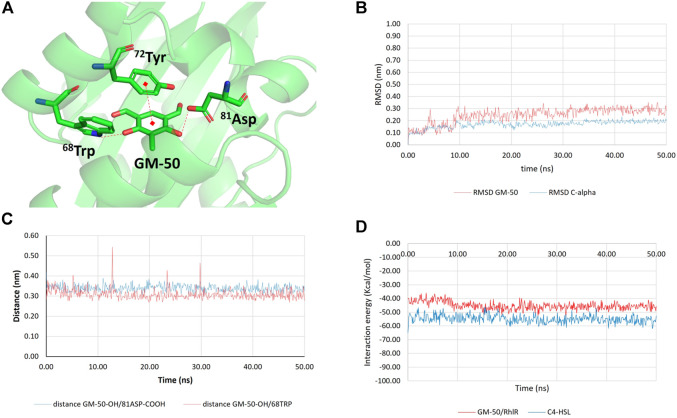
Results of molecular docking and molecular dynamics simulations between GM-50 and RhlR. **(A)** Predicted interactions between GM-50 and RhlR. H-bonds and π-π interactions are depicted as dashed red lines. **(B)** Root-mean squared deviation (RMSD) values for the protein C-alpha atoms (blue line) and GM-50 (displacement of ligand heavy atoms with respect to protein C-alpha atoms; red line). **(C)** Time dependent distance between GM-50 phenolic function and 81Asp sidechain (blue line) and between GM-50 phenolic function and 68Trp indole NH (red line). Both the distances are compatible with stable H-bonds. **(D)** Time dependent interaction energies (Kcal/mol) between GM-50 and RhlR (red line) and between C4HSL and RhlR (blue line).

Conversely, the complex GM-50/RhlR (Figure 8A) was stable for the entire simulation (50 ns; Figures 8B,C), confirming RhlR as the most probable biomolecular target. The interaction energy between GM-50 and RhlR (computed as the sum of the average short-range Coulombic and the short-range Lennard-Jones interaction energies between ligand and protein), measured throughout the simulation, remained very stable (−45.23 ± 3.00 kcal/mol; Figure 8D). For comparison purposes, the interaction energy between C4HSL and RhlR was measured, yielding a value of −54.83 ± 2.92 kcal/mol (Figure 8D).

## Discussion

*Pseudomonas aeruginosa* is an opportunistic pathogen able to cause infections, particularly in patients with chronic wounds, chronic respiratory tract diseases, urinary bladder catheters, or patients admitted to intensive care units (Vincent et al., 1995). The unique ability to establish persistent infections is often associated with the selection of multiple antibiotic-resistance strains. This observation strengthens the need for new drugs and therapeutic approaches to treat PA infections. QS signaling tightly regulates *P. aeruginosa* biofilm formation and virulence and plays a prominent role in establishing and maintaining chronic infections, thus representing a reasonable therapeutic target. Indeed, infections caused by *P. aeruginosa* whose QS communication systems have been disabled by mutation(s) are more readily cleared by the host organism than their wild-type counterparts (Bjarnsholt et al., 2005; Bjarnsholt et al., 2013). Moreover, molecules affecting the QS signaling system of *P. aeruginosa* promote infection clearing in different experimental models (Dong et al., 2007; Luo et al., 2017; Proctor et al., 2020). Although these compounds *per se* cannot eradicate bacterial infections they blunt pathogen aggressiveness by reducing microbial virulence, thus decreasing the severity of tissue damage and increasing the success of host immune defence and traditional antibiotic treatments (Soukarieh et al., 2018). Granting the rationale of QS inhibitors in *P. aeruginosa*, the identification of effective molecules has to face several drawbacks, including the complexity and the lack of structural data on critical elements of QS signaling in this pathogen.

Acyl-homoserine lactones (AHL)-dependent QS circuits, the Las and Rhl QS systems (Pearson et al., 1994), regulate the production of a variety of virulence factors and suppress the response of the host immune system. For these reasons, they have been targeted in several studies (Hossain et al., 2017; Ahmed et al., 2019; Piepenbrink et al., 2020). Thus, natural derivatives from plants, fungi, and marine organisms and synthetic molecules were tested as QS inhibitors (Kalia, 2013), even against *P. aeruginosa* (Yin et al., 2015; Chen et al., 2017). The mechanisms involved in QS inhibition by natural polyphenols are mainly based on antioxidant and antimetabolic effects whereas molecular docking analysis reported high binding affinity to the LasR domain mainly because of the electrostatic interactions of the natural compounds with the protein (Zhu et al., 1998; Teplitski et al., 2004; Ni et al., 2008; Chen et al., 2017). Moreover, natural polyphenols are extremely unstable molecules (Deng et al., 2018) and no one has yet been available for treatment. It has been reported that natural phenolic compounds from plants, such as eugenol, tea polyphenols, and thymol, possess a QS inhibitory activity preventing the production of virulence-associated factors and biofilm formation in *P. aeruginosa* (Ugurlu et al., 2016; Walsh et al., 2019; Antunes et al., 2021; Yang et al., 2021). Therefore, to identify active non-toxic molecules endowed with inhibitory activity on AHL-dependent QS signaling, we have screened a library of 81 phenolic derivatives generated in our laboratories. Through a two-step screening, namely AHL biosensors and quantitative RT-PCR, we identified a compound showing significant inhibitory effects on AHL-dependent QS-regulated genes *lasI/R*, *rhlI/R* and their mediated virulence factors *lasB* and *rhlA*. Analysis of the AHL profile by LC-MS confirmed the reduced release of the signal molecules in PAO1 exposed to GM-50 (Figure 3).

The assessment of the AHL activity showed that the anti-QS activity of the phenolic derivative may likely stem from the binding between the selected molecules and the receptor protein (Wagner et al., 2006). By combining *in silico* docking and molecular dynamics experiments, we demonstrated that GM-50 steadily binds Rhl receptor, while it is not able to stably bind other potential targets such as LasR, LasI, or RhlI. As previously reported, ^60^Trp_LasR_ is a key residue for either the positive or negative modulation of LasR activity (Gerdt et al., 2014). It is plausible that the analog residue ^68^Trp in RhlR may play the same regulatory role. Indeed, GM-50 established a lasting and strong H-bond with ^68^Trp through one of the phenolic functions. Other interactions that were predicted between GM-50 and the target protein are one H-bond between the ^81^Asp sidechain and the other phenolic function of GM-50, as well as a π-π stacking with ^72^Tyr sidechain. The binding mode remained stable for the entire 50 ns of simulations. For comparison, we also run in parallel the MDs simulation of the complex C4HSL/RhlR. The binding between the physiological ligand and the protein involved the interaction with ^68^Trp, further confirming the importance of this residue in RhlR activity regulation (Figures 7, 8). The comparison between the short-range (both Coulombic and Lennard-Jones) protein/ligand interaction energies suggests that GM-50 (E_interaction_ ∼ −45 kcal/mol) can bind the target protein in the presence of the activator (E_interaction_ ∼ −55 kcal/mol) but relative high concentrations of the phenolic inhibitor are required, suggesting that GM-50 still deserves structural improvement. For the sake of clarity, at this stage we have not computed the binding free energies (ΔG) but only the molecular mechanics interaction energies throughout the MDs simulations. Due to these observations, we predict that GM-50 preferentially binds RhlR, accordingly with the significant activity in reducing genes involved in the *rhl* pathway (Figure 1). Recent reports evidence the importance of rhl role in virulence, demonstrating that LasR-mutants frequently occur among clinical isolates. More than half of LasR-mutants isolates retain a LasR-independent RhlR activity (Groleau et al., 2021).

Inhibition of AHL-dependent QS signaling exerts favorable effects since down-regulating expression of several virulence factors such as pyocyanin, elastase B, and rhamnolipids will decrease the severity of tissue damage and increase the success of host immune defense (Turkina and Vikström, 2019). Two QS pathways control the production of pyocyanin in *P. aeruginosa*. Pyocyanin is a blue-green phenazine pigment able to interfere with the host cellular respiration, chelated iron absorption (Chang et al., 2014), and bacterial competitors (Diggle et al., 2003; Castañeda-Tamez et al., 2018). Elastase B is one of the major proteases produced by *P. aeruginosa*; it is directly involved in tissue damage and prejudicing immune responses (Husain et al., 2017). Rhamnolipids play a key role in biofilms formation and immune evasion, causing the killing of polymorphonuclear leukocytes and disrupting the mucosal epithelial barrier (Castañeda-Tamez et al., 2018). Our findings indicate that GM-50 is an active phenolic derivative in inhibiting pyocyanin, elastase B, and swarming motility accordingly to the dominant effect on the rhl-gene pathway of this molecule (Figures 1, 4). Indeed, GM-50 tested at 10 μM still reduced AHL-dependent gene expression, supporting the potential of this compound as an antivirulence agent in *P. aeruginosa* to counteract AHL-mediated QS systems (Figure 1C). Additionally, *P. aeruginosa*–secreted products may also affect epithelial cell phenotype impairing wound healing, directional migration (Ruffin et al., 2016), and production of inflammatory cytokines through activation of transcriptional factor kappa-B (NF-κB). The inhibition of QS with GM-50 significantly reduced the proinflammatory responses triggered by PAO1 on respiratory epithelial cells and potentially decreased tissue damage (Figure 5).

A strategic pathogenic mechanism for *P. aeruginosa* is the QS-regulated biofilm formation (Ciofu et al., 2015; Azam and Khan, 2019), which makes the eradication of the infection quite challenging and causes chronic inflammation in the underlying mucosa favoring antibiotic resistance (Chua et al., 2016). Thus, the weakening of biofilm by QS inhibitors facilitates antibiotic activity and is viewed as an attractive therapeutic strategy to improve the outcome of *P. aeruginos*a infections. In the present investigation, the biofilms were allowed to develop in Artificial Sputum Medium, a culture medium that mimics the components observed in chronic infections (Kirchner et al., 2012). Compared with control, phenolic derivative GM-50 did not result in direct biofilm inhibition in PAO1. However, we observed that in biofilm GM-50 possessed a synergistic effect with aztreonam, an antibiotic that we selected for its clinical relevance in the treatment of patients with chronic PA infections. Notably, the synergistic effects of GM-50 and aztreonam were observed on mature biofilm, the leading cause of nosocomial and chronic infection (Figure 4). Our results are in agreement with previous reports showing that QS inhibitors such as curcumin, itaconimides, and cinnamaldehyde enhance the activity of antibiotics such as ceftazidime, tobramycin, and colistin, respectively, on biofilm inhibition (Roudashti et al., 2017; Fong et al., 2019; Topa et al., 2020) but are poorly effective by themselves. Overall, the data available offer promising applications of QS inhibitors in combination with antibiotics to control biofilm-associated infections.

The *in vitro* results raise the question of what kind of effect our phenolic derivative might have on *P. aeruginosa* PAO1 virulence *in vivo*. Previous research showed that QS inhibitors block the virulence in *P. aeruginosa* in invertebrate model hosts, protecting *C. elegans, D. melanogaster*, or *G. mellonella* larvae (O’Loughlin et al., 2013; Papaioannou et al., 2013), and results correlate with mammalian acute infection models (Roudashti et al., 2017). In the insect models, *P. aeruginosa* accumulates and produces QS-dependent virulence factors, such as pyocyanin and elastase B, which are lethal. Compared with that in the untreated PAO1 group, in the *G. mellonella* model, our phenolic derivative GM-50 results in significant therapeutic potential (Figure 5). In a previous study, QS inhibitors such as hypertonic glucose and clofoctol were tested in *G. mellonella* larvae. The first one was injected at 200 mg/ml, a concentration higher than the concentration used for GM-50 in the present study (100 µM). Clofoctol was used at 100 µM, but it affects another PA QS-system, the Pqs, regulated by quinolones (D’Angelo et al., 2018; Chen et al., 2020). Since GM-50 did not alter bacterial growth rates or exert direct antimicrobial activity, our results suggest that inhibiting AHL-dependent Rhl circuits decreases pyocyanin, elastase B, rhamnolipids, and swarming motility. Moreover, GM-50 significantly affected the virulence of the bacteria and the host immune system and prolonged larval survival.

Finally, we tested the compound against PA clinical strains isolated from the respiratory tract of patients since it is well known that PAO1, a strain domesticated by extensive use in the laboratory, might produce results far from *in vivo* conditions for different virulence-related phenotypes (El-Shaer et al., 2016). In accordance with the genetic diversity and phenotypic heterogeneity of *P. aeruginosa* field strains (Finnan et al., 2004; Lebreton et al., 2021; Nassar et al., 2022), our clinical isolates significantly differed as regard pyocyanin production and swarming motility (data not shown). Although the clinical strains responded differently to treatment, overall, we reported a significant reduction in the production of pyocyanin, swarming and elastase B in most strains tested (Figure 6). The variable efficacy of QS inhibitor molecules on clinical strains is in agreement with previous studies investigating the ability of novel compounds to reduce virulence factors in clinical rather than in reference laboratory strains (García-Contreras et al., 2013; Guendouze et al., 2017). Indeed, clinical strains isolated from patients with chronic infections possibly accumulate mutations in the QS genes (Ciofu et al., 2010). The number of strains and virulence-related phenotypes that we considered in this study are not sufficient to drive a definitive conclusion. However, the strain-dependent response to the phenolic derivative is an issue that deserves to be taken into consideration when testing the anti-virulence properties of QS inhibitors, suggesting that therapeutic application of these molecules requires prior evaluation of activity on the isolate, likely for “traditional” antibiotics and in view of a personalized therapy.

**FIGURE 9:**
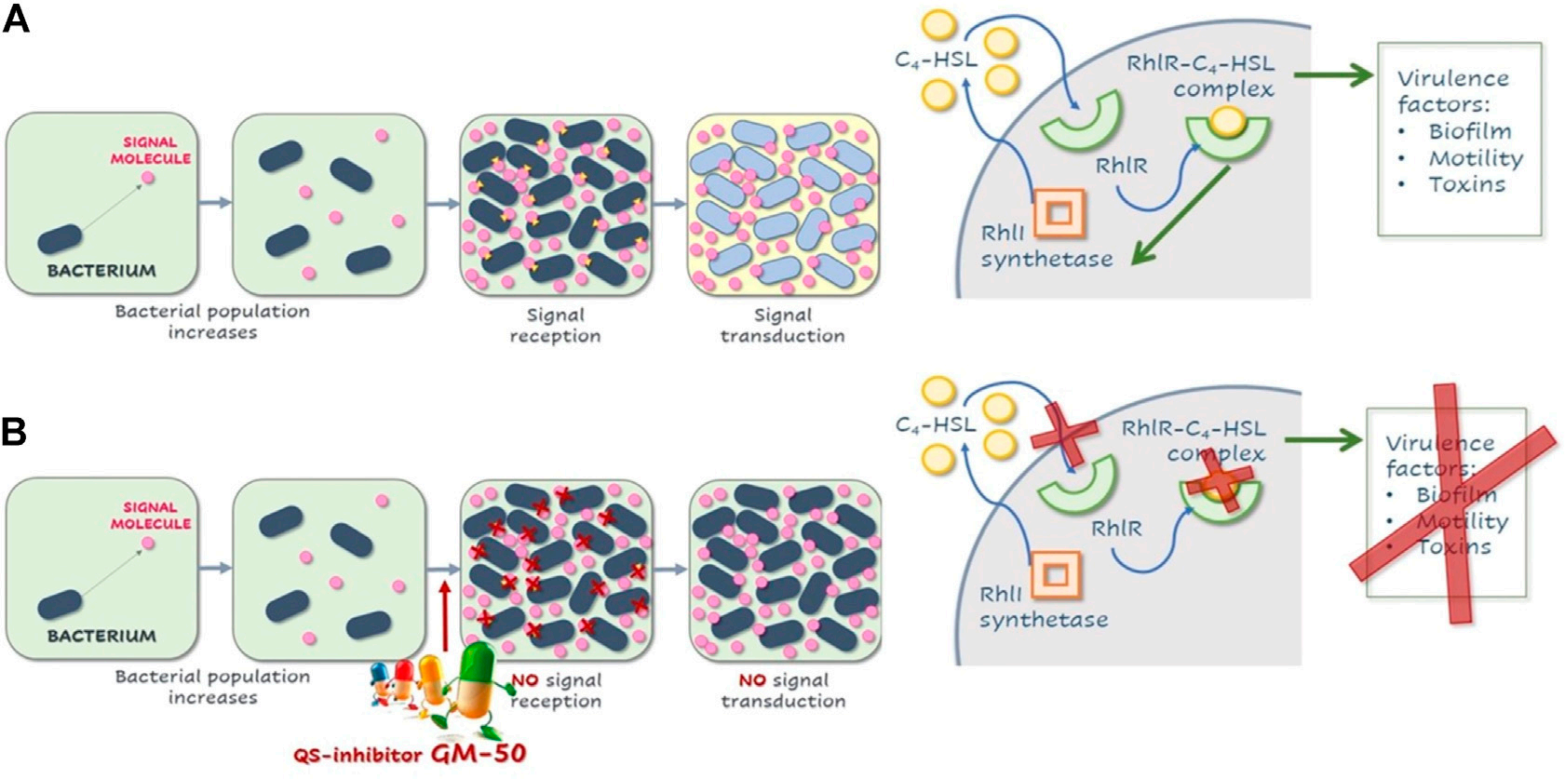
Schematic illustration of QS in P. aeruginosa and effects of GM-50. **(A)** Representation rhl quorum-sensing system in *P. aeruginosa*. After a threshold concentration of signal molecule C4HSL, the C4HSL-RhlR complex binds the promoter regions of responsive genes, activating or repressing their transcription. C4HSL-RhlR complex induces lasI expression, enhancing the production of C4HSL (autoinduction effect). Among virulence factors regulated by this pathway are genes involved in biofilm formation, pyocyanin and rhamnolipids release, elastase production, and swarming motility. **(B)** Compound GM-50 competes with C4HSL for the binding to the specific receptor, thus reducing the expression of *P. aeruginosa* virulence genes even in the presence of autoinducers in the environment.

In conclusion, the phenolic derivative GM-50, the most effective compound identified in this study, prevents AHL-dependent Rhl QS signaling and expression of virulence factors and enhances the anti-biofilm activity of aztreonam (Figure 9); it protects *Galleria mellonella* larvae and human A549 lung epithelial cells from *P. aeruginosa* induced killing and inflammatory damage. Molecular docking indicates that GM-50 interacts with RhlR, supporting the potential role for small-molecule modulators of quorum sensing as therapeutics. Moreover, since evidence for LasR-mutants in chronic infections points out the importance of the role of RhlR in infections, we expect that GM-50 represents a fascinating starting point to identify and develop a brand of new chemical classes of QS inhibitors active against *P. aeruginosa*.

## Data Availability

The original contributions presented in the study are included in the article/Supplementary Material, further inquiries can be directed to the corresponding author.
