# Supplementary material for: A novel phenolic derivative inhibits AHL-dependent quorum sensing signaling in Pseudomonas aeruginosa
Source: Front Pharmacol. 2022 Sep 20;13:996871. doi: 10.3389/fphar.2022.996871 (PMC9531014; doi:10.3389/fphar.2022.996871)
Supplement: Supplementary file 1 [file DataSheet1.PDF]

A novel phenolic derivative inhibits AHL-dependent Quorum Sensing signaling in *Pseudomonas aeruginosa*

Giulia Bernabè[1], Giovanni Marzaro[2], Giuseppe Di Pietra[1], Massimo Bellato[3], Anthony Pauletto[1], Melania Scarpa[4], Stefania Sut[2], Adriana Chilin[2], Stefano Dall'Acqua[2], Paola Brun[1*], Ignazio Castagliuolo[1*]

[1]Department of Molecular Medicine, University of Padua, Padua, Italy
[2]Department of Pharmaceutical and Pharmacological Sciences, University of Padua, Padua, Italy
[3]Department of Information Engineering, University of Padua, Padua, Italy
[4]Laboratory of Advanced Translational Research, Veneto Institute of Oncology IOV - IRCCS, Padua, Italy
*these Authors have contributed equally to the work and share the last authorship

Corresponding author
Paola Brun
Department of Molecular Medicine
University of Padova, Italy
E-mail: paola.brun.1@unipd.it

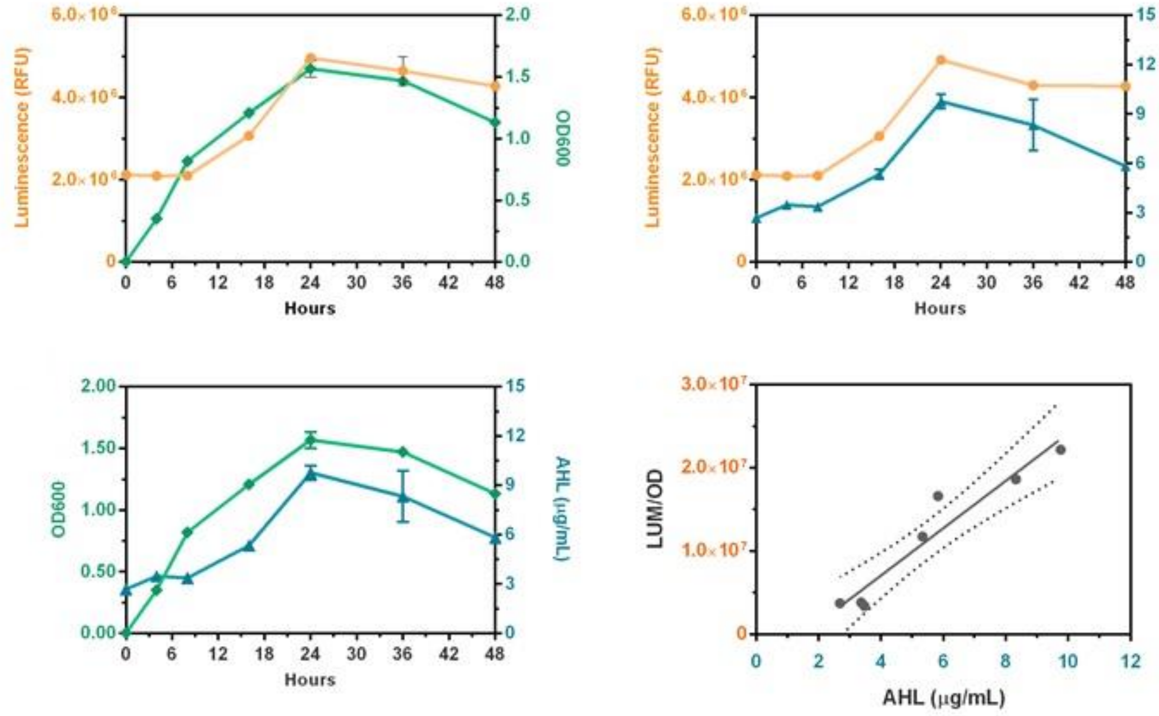

Supplementary Figure 1. The relationship between *P. aeruginosa* PAO1 and QS signal molecules. PAO1 (10^6CFU/mL) was cultured in LB, incubated at 37 °C and bacterial growth was monitored by measuring OD 600 nm. Culture supernatant was collected at specified time points and filtered. An aliquot was used to quantify the AHL profile by LC-MS technology. In parallel, 10 µl were added to *Vibrio harveyi* BB120 culture, and after 24 hrs *V. harveyi*-produced luminescence was measured. Correlation of *V. harveyi*-produced luminescence to AHL level in PAO1 culture media at different time points (0-12 hrs) was shown.

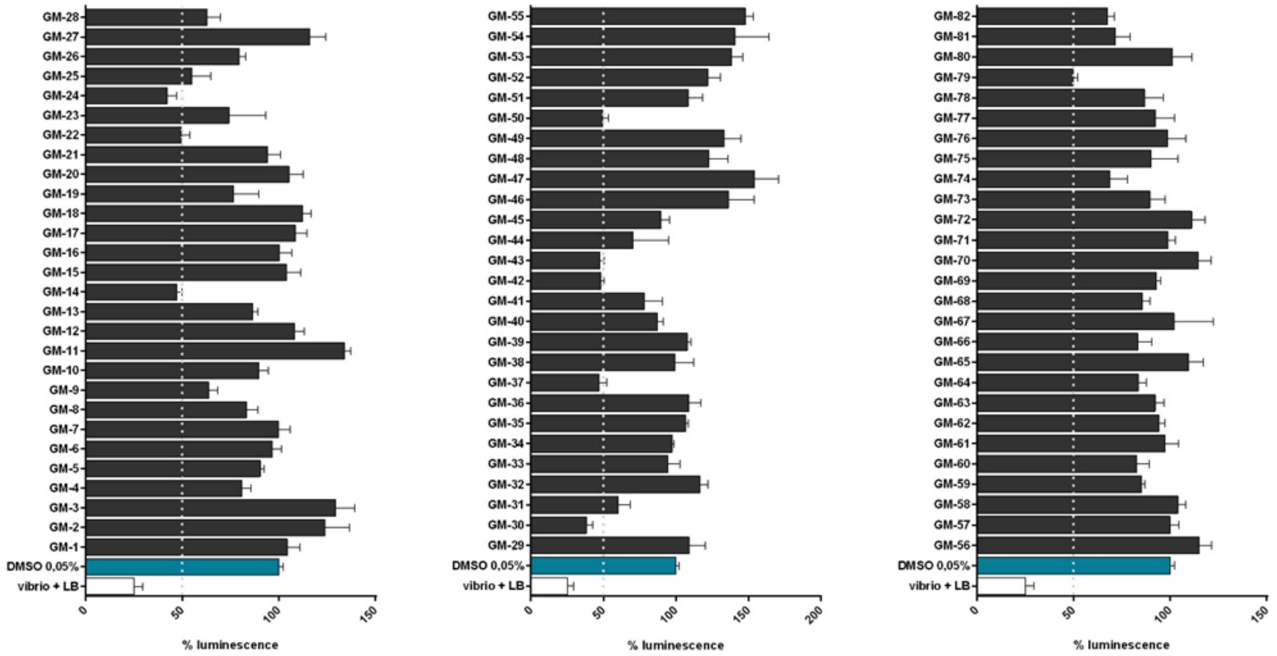

Supplementary Figure 2. Screening of 81 compounds by luminescence assay in *Vibrio harveyi* BB120. PAO1 (10^6 CFU/mL) were cultured in LB with compounds (100 µM) or vehicle (DMSO 0.05%) for 24 hrs. The culture supernatant was filter-sterilized and to evaluate AHL content, 10 µL were added to *Vibrio harveyi* BB120 cultures. Luminescence was quantified after 24 hrs. Luminescence obtained with PAO1 cultured with DMSO was arbitrarily assigned 100% AHL-production (each experiment was repeated at least three times with 5 replicates each).

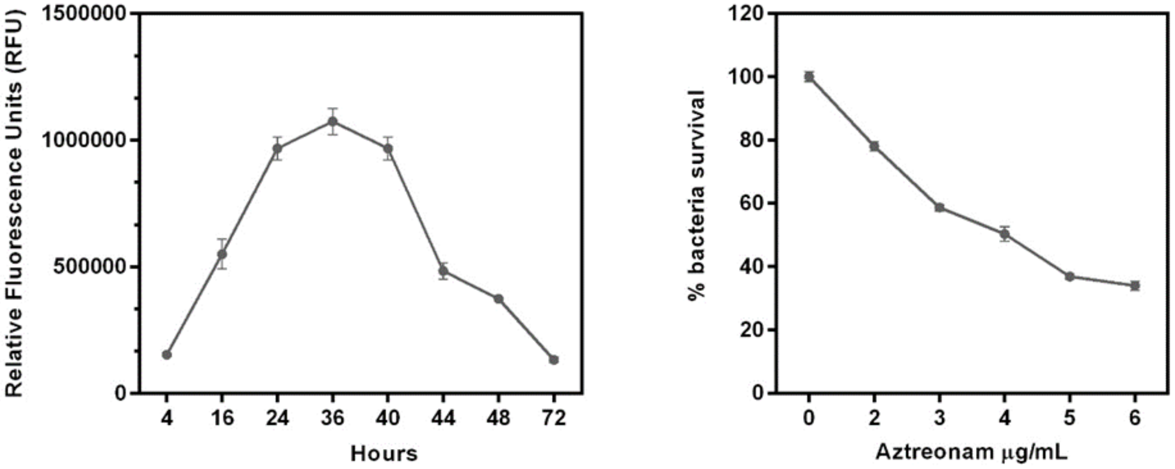

Supplementary Figure 3. Setting conditions for biofilm studies. PAO1 cultures were seeded in 24 wells and incubated at 37 °C with low agitation (75 rpm). Adhering cells were determined at the specified time points after incubation at 37 °C for 20 min with resazurin 0.01%. Biofilm formation was evaluated by measuring the relative fluorescence units (RFU) using a fluorimeter (Ex=530-570 nm, EM=590-620 nm). Data are reported as mean ± SEM (n=3). To determine $MBIC_{50}$ value, PAO1 biofilms were incubated with aztreonam at different concentrations. Biofilms were evaluated as described above. Data are reported as mean ± SEM (n=3).

1.1 Supplementary Tables

Compounds	% inhibition AHL-mediated bioluminescence
GM-14	52,6
GM-22	50,4
GM-24	57,8
GM-30	60,1
GM-37	52,8
GM-42	51,8
GM-43	52,5
GM-50	50,5
GM-79	50,2

Supplementary Table 1. Percentage of inhibition of AHL-mediated bioluminescence in *V. harvey* BB120 treated for 24 hrs with PAO1 supernatants treated or not with compounds 100µM for 24 hrs.
